# Application of Microorganisms for the Valorization of Side-Products of Rapeseed De-Oiling

**DOI:** 10.3390/biom15070917

**Published:** 2025-06-22

**Authors:** Michal Jacek Binczarski, Justyna Zuberek, Justyna Fraczyk, Beata Kolesinska, Milivoj Radojčin, Ivan Pavkov, Ewa Wiktorowska-Sowa, Jan Piotrowski, Zbigniew Jerzy Kaminski, Izabela Alina Witonska

**Affiliations:** 1 Institute of General and Ecological Chemistry, Faculty of Chemistry, Lodz University of Technology, Zeromskiego 116, 90-924 Lodz, Poland; justyna.zuberek@p.lodz.pl (J.Z.); zbigniew.kaminski@p.lodz.pl (Z.J.K.); izabela.witonska@p.lodz.pl (I.A.W.); 2 Institute of Organic Chemistry, Faculty of Chemistry, Lodz University of Technology, Zeromskiego 116, 90-924 Lodz, Poland; justyna.fraczyk@p.lodz.pl (J.F.); beata.kolesinska@p.lodz.pl (B.K.); 3 Faculty of Agriculture, University of Novi Sad, Trg Dositeja Obradovi’ca 8, 21000 Novi Sad, Serbia; milivoj.radojcin@polj.edu.rs (M.R.); ivan.pavkov@polj.uns.ac.rs (I.P.); 4 National Food Industry Group, 87-100 Torun, Poland; ewa.wiktorowska-sowa@kgssa.pl (E.W.-S.); jan.piotrowski@kgssa.pl (J.P.)

**Keywords:** microbial valorization, rapeseed byproducts, sustainable agriculture, waste management, *Brassica* seeds

## Abstract

The increasing demand for sustainable agriculture and environmental protection has prompted the exploration of innovative methods to valorize byproducts from rapeseed oil production. This review focuses on the application of microorganisms as a promising approach to transforming rapeseed de-oiling residues, such as cake and meal, into valuable products. This review discusses traditional and modern methods of rapeseed oil extraction, the composition and challenges posed by rapeseed byproducts, and the presence of antinutritional components such as glucosinolates, erucic acid, and phytic acid. Microbial applications, including the production of industrial enzymes, enhanced digestibility, and the neutralization of antinutritional factors, are examined as key solutions for waste valorization. Additionally, the role of microbial consortia and genetic modification in optimizing transformation processes is discussed. This review underscores the potential of microorganisms in creating eco-friendly, scalable technologies that contribute to resource efficiency and environmental sustainability in the agricultural and biotechnology sectors.

## 1. Introduction

Holistic utilization of agriculture products is nowadays a well-established approach to environmental protection. Progress in this area is reached through the implementation of more advanced technologies and less expensive procedures leading to the transformation of waste materials into valuable products. As yet, the potential engaged in utilization of the side-products accompanying the production of many key crops has not been fully explored [1]. An ample example of the benefits arising from the synchronized expansion of main crop volume with significant progress in the valorization of accompanying the side-products commodities is rapeseed oil production [2]. The massive farming scale, genomic variation in cultivated *Brassica* oil crops, diversified properties, components and recommended procedures used for oil isolation generate a range of assorted removable side-products. Their successful valorization requires a palette of technological procedures [2]. One of the most promising strategies is based on the application of microorganisms. The comprehensive chemical transformations supported by microorganisms cells proceeds according to a sustainable economy, under mild conditions, promoting the formation of diversified final products and upscaling of processes is relatively simple [2,3]. Moreover, the outcome of microbial processing (transformation) can be additionally modulated by selection of the microbiota strain, genetical modification and/or application of microorganisms consortia [4,5].

The genus *Brassica* includes 73 accepted species [6,7,8], but only four are commonly cultivated for oil production: *Brassica napus* (Swede rape), *B. rapa* (turnip rape), *B. juncea* (Indian mustard), and *B. carinata* (Ethiopian mustard) [9]. *B. napus* is the most widespread in temperate regions of the northern hemisphere. *B. rapa* has the oldest cultivation history in Europe, Western Asia, and Northern Africa. *B. juncea* is mainly grown in India and some areas of China, while *B. carinata* is primarily cultivated in Ethiopia [10]. Rapeseed was already grown in India around 4000 BC and in Europe by 2000 BC [10,11].

Breeding and selection of *B. rapa* in both East Asia and Europe have led to phenotypically diverse varieties. In Europe, oilseed forms were selected from the turnip type *B. rapa* in the 14th century [12].

Breeding and selection of *Brassica rapa* in both East Asia and Europe have produced a wide range of diverse varieties. In Europe, oilseed forms were selected from the turnip type of *B. rapa* as early as the 14th century [12]. *B. rapa* (2n = 20, genome AA) has also played a key role in breeding programs, as it is one of the ancestral species of the allotetraploids *Brassica napus* (2n = 38, genome AACC) and *Brassica juncea* (2n = 36, genome AABB). It has also been used to develop synthetic allohexaploid *Brassica* species [10]. The most important oilseed crop, *B. napus*, is an allopolyploid formed by hybridization between two diploid species: *B. rapa* (AA, n ¼ 10) and *B. oleracea* [13,14,15].

Comprehensive genomic data for rapeseed are available. Raw sequence data can be accessed through public repositories such as the Sequence Read Archive at the National Center for Biotechnology Information and the European Nucleotide Archive. Additionally, an online rapeseed genomic variation database provides global access to single-nucleotide polymorphisms (SNPs) and insertions/deletions. This database [16] currently includes 34,591,899 high-quality SNPs and 12,281,923 high-quality insertions/deletions across 1007 rapeseed accessions from around the world, along with search tools for exploring gene annotations and variations [17,18].

The rapeseed crop is pods with 5–10 cm siliques containing 20–30 seeds of diameter 1.5–3 mm each. Mature seeds are hard, round and black or yellow colored. Each of them consists of a seed coat surrounding a reduced number of endosperm cells and the embryo, composed of cotyledon, hypocotyl, and radicle. The oily bodies and protein bodies essential for germination of the seeds are located in the embryo [19,20,21,22].

For many years, rapeseed was grown on a limited scale due to the presence of several antinutritional compounds. The most significant were: (i) a high erucic acid content (up to 54%) in the oil, which is harmful to heart muscle; (ii) glucosinolates, which break down into toxic and irritating substances when seeds are mechanically damaged; and (iii) other antinutritional factors. A major breakthrough came with the development of low-antinutrient varieties of *Brassica napus* and *Brassica campestris* using traditional breeding techniques. These varieties, known as “canola” in Canada or “00 rapeseed cultivars” in Europe, contain less than 2% erucic acid and under 30 μmol/g of glucosinolates [23,24].

Today, rapeseed is the second most important oilseed crop in the world after soybean, with global production exceeding 100 million metric tons. It is the leading oilseed in temperate climates [25,26]. Demand for canola oil is expected to grow by 40% by 2025. The largest producers are the European Union, followed by Canada and China. Rapeseed oil is widely used both as an edible oil and as a raw material for processed products such as margarine, detergents, and biodiesel (EUBIA Biodiesel market) [27].

The fatty acid composition of rapeseed oil is well known, and its metabolic effects have been described [28,29]. The distinct flavor profile of virgin rapeseed oil has also been studied, with up to 47 key aroma compounds identified using gas chromatography/mass spectrometry, electronic nose technology, and sensory analysis [30]. Additionally, a link between 144 components of rapeseed oil and cake with the petal color of the plant was discovered [31]. Finally, the ecological impact of using rapeseed oil as a biofuel has been evaluated, including comparisons of greenhouse gas emissions and energy payback times with other crops such as corn, sugarcane, and soybean [32].

**Figure 1:**
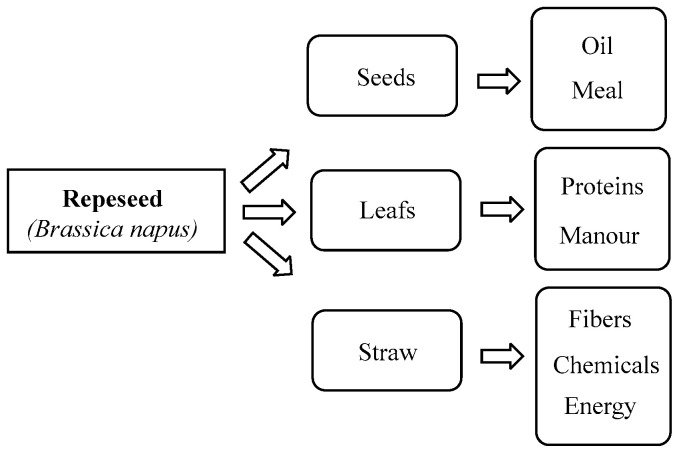
Main products of rapeseed cultivation [21,31].

According to the Food and Agriculture Organization of the United Nations, global rapeseed production and the cultivated surface have grown continuously over the past 40 years, rising in recent years above 36 million hectares, respectively, in 2019 [33]. The high volume of rapeseed oil production generates a huge amount of side-products known as meal or cake obtained after oil isolation from the rapeseed and residual plant material remaining after seeds harvesting (Figure 1). In Europe, the winter oilseed rape yield 3 and 4 tons of seeds per hectare and 3 to 10 tons of stalks. Thus, the seeds represent 28–50% of total biomass, and the remaining crop residues represent 50–72% of total biomass [34,35,36,37].

Particular attention is paid to residual material obtained after oil isolation from rapeseed (named cake and meal) due to its very high nutritional value. The price of rapeseed meal in 2019/2020 reached 243 USD/ton [38]. Their successful utilization and valorization are vital, due to economic reasons as well as the obligation to protect the environment. Several technologies developed to reach the goal are recently available. The total costs of valorization procedures depend on many factors including rapeseed cultivar, agriculture, climate conditions, the de-oiling procedure and finally technology applied to resolve problems caused by the waste materials. Numerous advanced and efficient procedures for rapeseed waste management are recently offered. Herein, we focus on the application of microorganisms for rapeseed waste transformation expecting this approach, could be the most environmentally friendly [39].

Despite the increasing availability of rapeseed meal and other Brassica byproducts, their full valorization remains limited due to the presence of residual antinutritional compounds and the complex composition of these materials. At the same time, growing interest in sustainable protein sources, circular bioeconomy, and functional food ingredients highlights the need for effective biotransformation strategies. This review aims to explore the potential of microbial transformation as an efficient and sustainable approach for improving the nutritional and functional quality of rapeseed-derived materials. We focus particularly on how the use of selected microbial strains or consortia can help degrade undesirable components and enrich the final product with value-added metabolites. The findings contribute to the development of innovative, eco-friendly technologies for the processing of oilseed byproducts, supporting both waste reduction and the production of high-value functional ingredients for food and feed applications.

## 2. Procedures for De-Oiling Rapeseed

Applied procedures of oil extraction determine the value, properties, composition and scope of application of isolated oil, as well as residual material (pomace), reach in proteins. Seeds are the most gently treated by using expeller processing in the procedure known as cold-pressing, also termed double pressing [26,40]. The isolated cold-pressed rapeseed oil offers health benefits due to its preserved fatty acid profile and bioactive compounds including tocopherols, phenolic compounds, phytosterols, and carotenoids [26,41]. These components of cold-pressed rapeseed oil regulate the blood lipid profile, insulin sensitivity, and glycemic control, as well as offer antioxidant and cytotoxic activity. The resulting cake has a higher nutritional value due to residual oil content, which can range from 8% to 11% [42].

**Figure 2:**
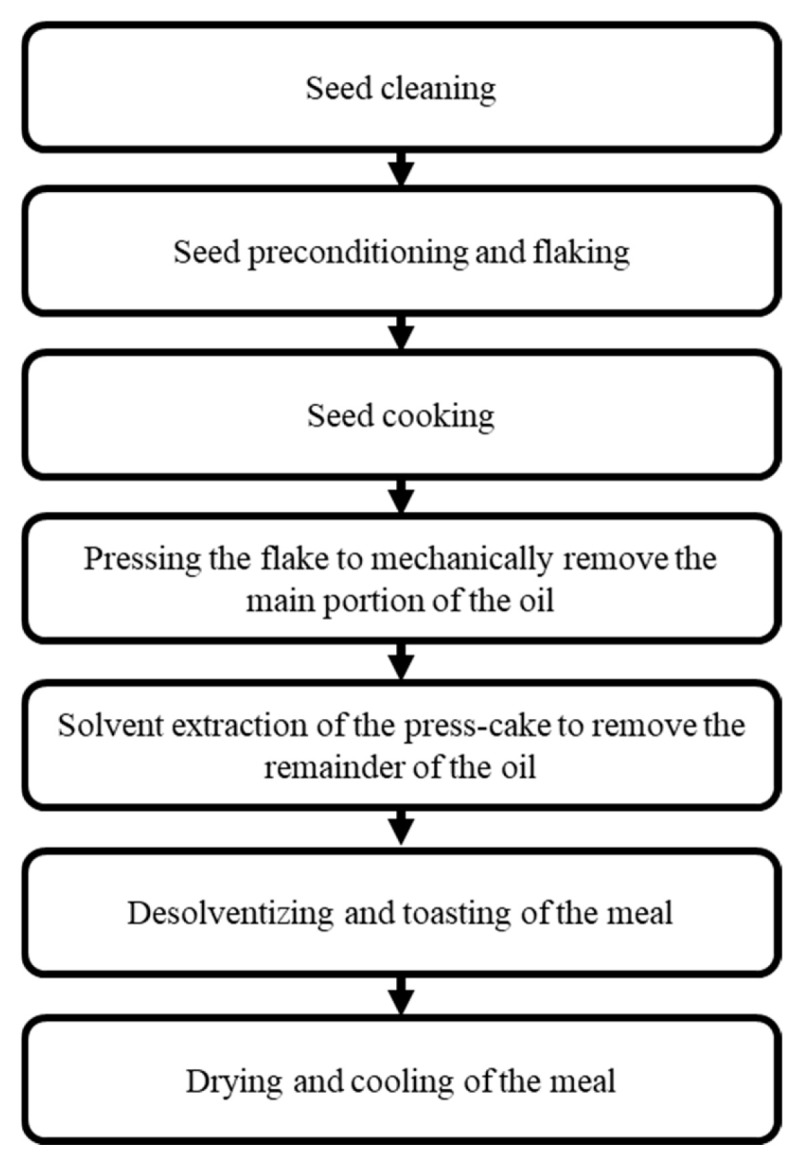
Typical technological operations are used in the process of oil isolation from rapeseed.

To obtain more oil out of rapeseed, factories can add extra steps to their usual process. These extra steps are often more intense or ‘harsher’—for example, they might use high heat or chemicals, unlike simple cold pressing. How the factory actually carries out these extraction steps (like the temperatures used or the chemicals involved) really matters. It directly affects how good the final oil will be, and also the quality of the leftover solid part, called ‘meal’ (which is used for animal feed). The most typical way to obtain oil from rapeseed involves a series of steps, one after another. You can see these steps laid out in Figure 2 [43].

Seed cleaning operation, removing undesired, docked materials before to processing, also known as “destoning”, is obligatory to any one technology and in all cases enhances the quality of final products. A preconditioning stage is employed with an injection of live steam to attain the desired moisture of the seed preventing its shattering during flaking [44]. In the flaking operation, the softened seeds are passed through two contra-rotating smooth cylinders adjusted with a distance ranging from 0.25 to 0.4 mm. The optimal thickness of the flakes ranges between 0.30 and 0.38 mm. Flattening the seeds ruptures the seed coat and the lipid-containing dolesome organelles in cotyledons. Then, flakes are heated to 80–100 °C in 2 or 3 steam-heated drums known as cookers to reduce the moisture content and oil viscosity, thereby promoting the coalescence of oil droplets, and enhancing pressing efficiency and oil quality [45]. Cooking also contributes to the feature of oil and cake by minimizing the sulfur content responsible for undesirable smells when the oil is used for cooking and poisoning the catalysts used in the hydrogenation of the oil [46]. Cooked seeds are mechanically pressed in a screw press to remove approximately 70–89% of their oil content and to obtain the residual mass known as a cake [47].

The remaining cake is dried, extruded into smaller pieces and extracted with the appropriate solvent to obtain additional 15–20% oil. After removing the solvent (usually hexane), the proteins reach the residual material obtained, known as a meal [45]. At this stage of the process, for each 1 ton of meal, approximately 1 kG hexane is lost to the atmosphere. Recently, in practice, most hexane is replaced with a mixture of 2-methylpentane, and 3-methylpentane [47]. To further reduce the environmental impacts of hydrocarbon, and improve oil yield and quality, novel alternatives [25] were currently proposed such as extraction with supercritical CO_2_ [48], aqueous enzyme extraction [49,50], microwave and ultrasound processing [51,52].

Increased process temperature deteriorates the nutritional value of the meal and rapeseed oil [53]. At 140 °C, for 5 min, α- and γ-tocoferol contents decrease by 41% and 36%, respectively, observed. Heating to 100 °C for 7 min also lowers α- and γ-tocoferol content by 36% and 34%, respectively [53,54].

The elevated temperature causes changes in color and the smell of the meal, decreases lysine content, reduces the bioavailability of proteins and stimulates the formation of antioxidants [53,55]. Moreover, it was found that the volatile compounds defining the sensory quality of virgin oil depend on the profile of microorganisms populating rapeseed during storage. By DNA sequencing, bacteria species were identified and 29 strongly flavored volatile compounds produced by them were identified by solid-phase microextraction followed by gas chromatography–mass spectrometry. Most of the identified compounds were products of fatty acid degradation such as alkanes, alkenes, aldehydes, ketones and alcohols, but also terpene, pyrazines and sulfur-containing organic compounds [56]. Table 1 connects specific bacteria isolated from rapeseed with the volatile organic compounds (VOCs) they produce by metabolizing fatty acids, proteins, and phytochemicals in rapeseed residues. This synthesis is based on the identified bacterial strains and their associated VOCs from relevant studies [56,57,58,59].

**Table 1:** Bacteria isolated from rapeseed with volatile organic compounds.

Bacterial Genus/Species	Key Volatile Compounds Produced	Compound Class/Origin	Notes	Ref.
*Pseudomonas* spp.	Alkanes, alkenes, aldehydes, ketones, alcohols	Fatty acid degradation products	Common rapeseed colonizers producing typical off-flavor compounds	[57,59]
*Bacillus* spp.	Sulfur-containing compounds (e.g., dimethyl disulfide), pyrazines	Sulfur volatiles, Maillard reaction products	Contribute to pungent and nutty aromas; pyrazines formed during seed roasting and storage	[59]
*Microbacterium* spp.	Aldehydes (hexanal, nonanal), ketones	Lipid oxidation products	Associated with oxidation of unsaturated fatty acids	[56,57]
*Staphylococcus* spp.	Alcohols, aldehydes, ketones	Fatty acid and amino acid metabolism	Influence aroma profile through diverse VOCs	[58,59]
*Enterobacter* spp.	Sulfides, nitriles	Glucosinolate degradation products	Linked to pungent aroma via GLS breakdown	[57,58]
*Pantoea* spp.	Pyrazines, furans	Maillard reaction products	Important for nutty and roasted aroma notes	[56]

Analysis of fermented rapeseed meal using an electronic nose system and headspace solid-phase microextraction, gas chromatography, and mass spectrometry revealed the loss volatile hydrocarbons and some aldehydes and slowly increased the concentration of tetramethylpyrazine, butanoic acid, benzenepropanenitrile, 2-methylbutanoic acid, trimethylamine, 2,3,5-trimethyl-6-ethylpyrazine, and 2,3,5-trimethylpyrazine. This strongly confirms that treatment with *Bacillus subtilis* and *Actinomucor elegans* can significantly modify the flavor and palatability of rapeseed products [60].

## 3. Cake and Meal

Cake and meal, byproducts obtained after oil isolation from rapeseed, are available in powder, patch and granular forms. Their energy value is variable and depends on the amount of residual oil. The protein content reaches 35–40% with high levels of exogenic lysine, methionine and tryptophan. The essential amino acid profile and protein utilization efficiency in human subjects show that canola can be ranked as a source of high-quality protein, comparable with milk and egg proteins [61,62,63]. However, its nutritional value is decreased by the presence of non-protein components such as undigestible carbohydrates (fibers), phenolics derivatives, and phytates. Thus, the application of meal and cake for nourishing requires additional treatment removing antifeedants.

The main protein components of the residual product obtained after rapeseed de-oiling are a storage globulin known as cruciferin, constituting approximately 60% of the total protein pool, napin classified to the albumins family, sharing 25% of the protein pool, and oil body proteins (15%).

**Figure 3:**
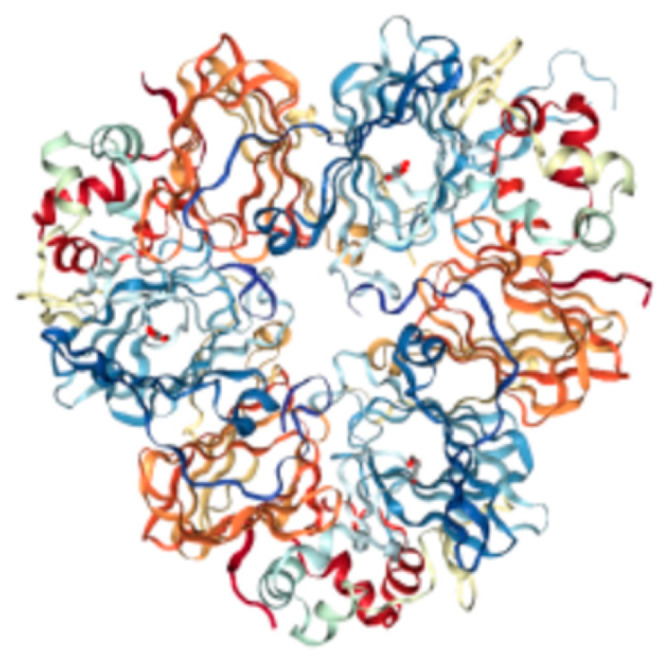
Procruciferin structure determined by crystallographic studies. Image of PDB entry 3KGL [69,70].

**Figure 4:**
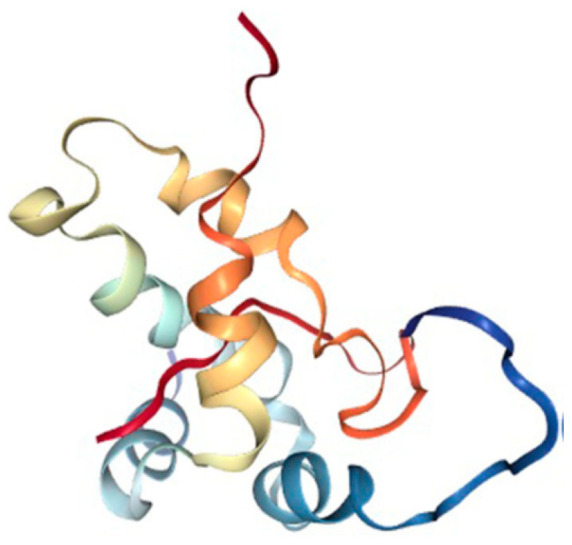
Procruciferin The structure of napin [71].

Cruciferin was identified both in cold-pressed and pre-press solvent-extracted meals either intact or in the form of separated α- and β-subunits [64]. Polypeptide α is built from 254 to 296 amino acids (approximately 40 kDa), whereas polypeptide β is composed of 189–191 amino acids (approximately 20 kDa). Both chains are covalently linked by disulfide bonds and after transporting to protein storage vacuoles, assembled into hexamers (Figure 3). Due to intensive post-translational modifications, cruciferin was identified in several iso-forms [65]. In rapeseed, cruciferin serves as a reserve of amino acids and nitrogen for germinating embryos [66,67,68]. The less abundant component of the rapeseed protein pool is napin (Figure 4), belonging to the prolamin superfamily.

The structure of mature napin consists of one short- (4 kDa) and a long- (9 kDa) peptide chain linked together by two inter-chain disulfide bonds. The long chain also contains two additional intra-chain disulfide bonds. These stabilize the secondary structure up to 75 °C. Several isoforms of the protein were identified [66,72].

The third and the least abundant, but most diversified, group of rapeseed proteins consists of oil body proteins [73]. Their structures resemble surfactants with long hydrophobic domains and hydrophilic heads stabilizing oil droplets in strongly polar aqueous cytosolic environment by encapsulation of hydrophobic neutral lipids into small hydrophilic particles [74]. The predominant component *Brassica napus* oil body proteins (up to 90%) are oleosins possessing molecular mass 18–25 kDa [75]. The other identified proteins are a group of caleosins with a molecular mass of approximately 30 kDa capable to bind calcium, and steroleosins with a molecular mass of 40 kDa [76]. The review article discussing structure and function of oil bodies proteins is available [73]. Recently, isolation procedures, effects on oil extractability, emulsifying and foaming properties of rapeseed proteins are intensively studied [77,78,79,80].

Whole canola seed or crude de-oiled seed (meal) is rarely or not used as a source of food protein due to the presence of antinutrient components even in recently dominated rapeseed cultivars “canola” or “00” variety [38,81,82,83]. Their nutritional value depends on multiple factors, with the most important being: rapeseed crop variety, the farming and oil pressing procedure (parameters), determining the content of antinutritional components as well as bioavailability of nutritional factors [64,84]. By application of a defined procedure, it is possible to extract from the crude meal the most undesirable components or alternatively, isolate a high nutritional value protein concentrate with a well-balanced amino acid profile. It is also possible to isolate protein products selectively enriched with cruciferin or napin [82,85,86].

## 4. Antinutrient Components

Reach in protein and lipids *Brassica* seeds is a highly attractive foodstuff for numerous pests. To protect the seeds and deter the pests, all members of the *Brassica* family use a complex arsenal of defensive compounds. The presence of the antinutrient components including glucosinolates, synapine, tannins, phytic acid and fibers substantially reduce the consumption value of row rapeseed [81,82,87]. The typical canola meal incorporates 1.0% sinapine, 2.3% phytic acid, 4.2 mm/g glucosinolates and 32.4% total dietary fiber [23].

Four strategies are applied to minimize the undesirable effects caused by the presence of antinutrient components limiting the application of canola meal as food and forage components. These are based on the application of physical methods, chemical methods, microbial methods and genetic breeding methods. The first three lead to the development of technology used for rapeseed processing. Efforts developing the breeding methods are continued, expecting its further potential after the most spectacular achievements obtained by the selection of Canola or “00” cultivar offering the crop with reduced content of erucic acid and glucosinolates and “000” cultivar with additionally reduced undigestable fiber content. Meal obtained from yellow-seed compared to typical black-seed product is characterized by higher protein content: 35.46% compared to black seeds (30.29%), higher sucrose 7.85% (vs. 7.29%), dietary fiber lowered to 26.19% (vs. 34.63%) and crude fiber lowered to 4.56% (vs. 8.86%), and glucosinolates lowered to 22.18 (vs. 28.19 mol/g). Also,, phytic acid and total polyphenols decreased to 4.98% and 2.67%, respectively, as compared to the black seed cultivar with 5.60% and. 2.82% (Table 2) [38,88,89,90]. The careful characterization of metabolic profiles of various yellow- and black-seed rapeseed showed substantial differences in 54 flavonoids, 24 glucosinolates, 65 lipids 31 phenolic compounds and other 74 polar components [91,92]. There is however information reported that seeds of yellow cultivars are less resistant towards mechanical damage, pathogenic fungi and prolonged storage [93].

**Table 2:** Meal obtained from yellow seeds compared to typical black seeds.

Type of Components	Meal Obtained from Yellow Seed [%]	Meal Obtained from Black Seeds [%]	References
Protein [38]	35.46	30.29	[38]
sucrose	7.88	7.29	[38,94]
dietary fiber	26.19	34.63	[95]
crude fiber	4.56	8.86	[89]
Glucosinolates	22.18	28.19	[88,89]
phytic acid	4.98	5.60	[38,89]
total polyphenols	2.67	2.82	[38,89,90]

### 4.1. Glucosinolates

**Scheme 1:**
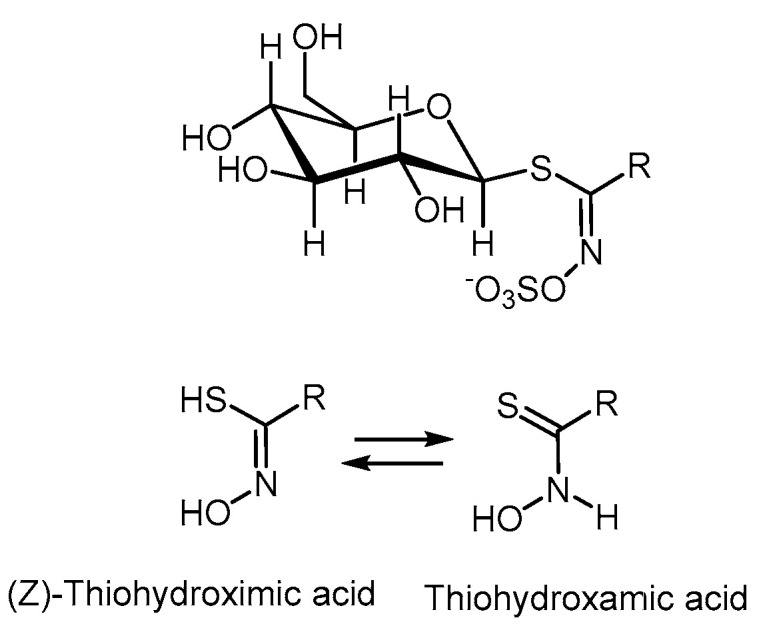
Glucosinolates are esters of (Z)-thiohydroximic acid anomerically connected to S-β-D-glucopyranose sulfated on the nitrogen atom of aldoxime function.

The glucosinolate–myrosinase couple is a powerful chemical defense system found in plants of the *Brassicales* (*Capparale*) order, activated after wounding of the plant tissue [96,97,98]. Glucosinolates consist of a class of relatively stable secondary metabolites located inside the parenchymatous tissues of plants, identified in hypocotyl, cotyledons, leaves, stem, roots, seedlings and also in the medium supporting the roots [99,100]. Their structures and abundance in plant tissue of individual glucosinolates vary appreciably with the development of the plant [101]. Formally, their structure is formed by acylation of S-β-D-glucopyranose with O-sulfated (Z)-thiohydroximic acid [102]. In contrast to other secondary metabolite defense systems, glucosinolates are not toxic. They are water soluble and classified to the glucosides family or more precisely to glucose esters of N-sulfated (Z)-thioxydroximic acid (Scheme 1). The nature of substituent R, derived directly from amino acids side-chains or formed by their further biochemical transformations, diversifies the glucosinolate structures [103,104].

The attachment of strongly polar sulfate group and glucosidic fragment to usually lyophilic substituent R with a tendency to subsequent multi pathways degradation created severe problems with their isolation and identification [105,106]. For different chromatography systems, the factors influencing the retention and selectivity of several natural and artificial nonsulfated glucosinolates have been studied. The most general method for analysis of glucosinolate mixtures was found HPLC on different apolar or weakly polar stationary phases with gradient elution [107] and the direct analysis of HPLC fractions by coupled thermospray mass spectroscopy [108]. Alternative analytical methods for determining the glucosinolate content involved myrosinase immobilized on the membrane. A comparison of data obtained with the biosensor and those from HPLC indicated a good correlation. Moreover, immobilized myrosinase was stable and retained its activity after approximately 1000 assays [109]. Glucosinolate diversity within a phylogenetic framework of the tribe *Cardamineae* (*Brassicaceae*) studied by HPLC-MS/MS and NMR-based analysis identified 70 desulfoglucosinolates [110]. For a collection of 31 glucosinolates and desulfoglucosinolates representing 17 different side-chains, accurate descriptions of the ^1^H-, ^13^C-, and ^15^N-NMR parameters is available. The chemical shift assignments were supported by 2D COSY, HSQC, and HMBC spectra [105].

According to the most recent critical review, the number of glucosinolates isolated from plants and identified is estimated on somewhere between 88 and 137 [111]. All proposed biosynthetic pathways leading to glucosinolates starts from amino acids. Fragment R of glucosinolates consists proteinogenic amino acid side-chain. Its further modification, by introducing additional functional groups or side-chain elongation, is already well documented [112]. The variation in the side group is responsible for the variation in the biological activities of these plant compounds [96]. The recently proposed semisystematic naming (nomenclature) of glucosinolates consists of the chemical name of that side-chain followed by “glucosinolate”. Spelling glucosinolate names are accepted in one or two words. Both forms have equivalent meaning (e.g., benzylglucosinolate equivalent to benzyl glucosinolate) [111]. Considering the character of fragment R it is possible to identify the two groups of glucosinolates namely aromatic and aliphatic [113]. Both groups of glucosinolates are prone to degradation to the sharp and pungent testing isothiocyanates. Activation occurs upon wounding, (e.g., due to chewing) which brings glucosinolates in contact with the hydrolytic enzyme—myrosinase (EC 3.2.3.147) [114]. This initiates splitting of the thioglucosidic bond and the formation of glucose and an unstable aglycone—thiohydroximate-O-sulfate.

**Figure 5:**
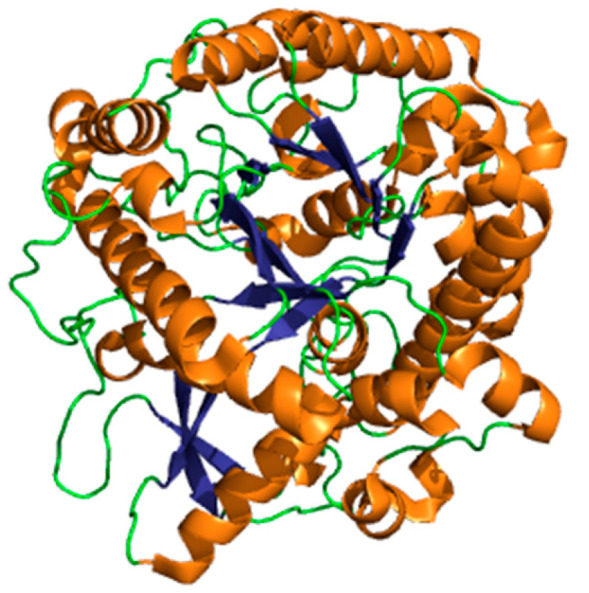
Myrosinase from *Sinapis alba*.

**Scheme 2:**
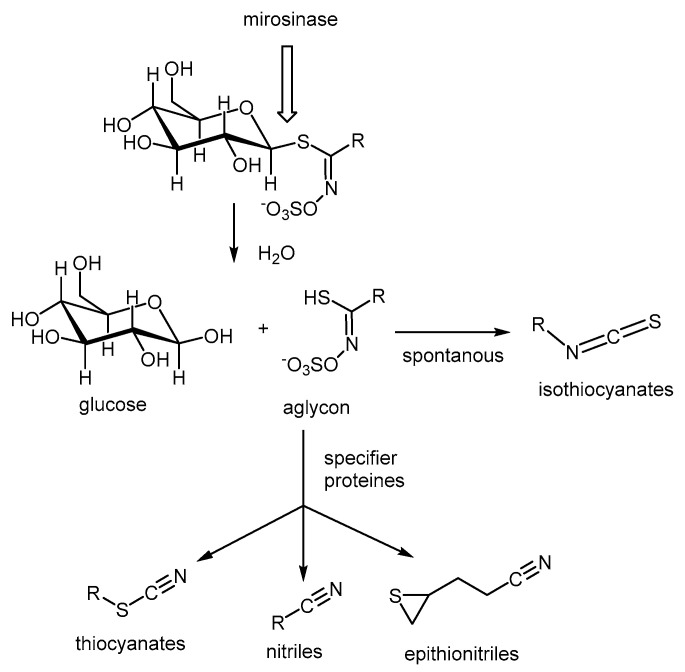
Degradation pathways of glycosinolates.

Its further transformation proceeds spontaneously and leads to isothiocyanates. The non-enzymatic pathway of glucosinolates splitting with the formation of isothiocyanates as dominating products was also observed at low pH and in the presence of ferric ions Fe^+2^ [115]. In both cases, the glucosinolates degradation (Scheme 2) leads to non-palatable products. Moreover, myrosinase if not destroyed, will increase the break of glucosinolates into their toxic metabolites in the digestive tract [25]. Their interactions with consumer organism depends on the structure of R group. Some glucosinolates in *Brassica* species produce sulforaphane, phenethyl, and indolylic isothiocyanates that possess well-documented beneficial anticarcinogenic activity. On the other hand, progoitrin which is dominating component in a palette of rapeseed glucosinolates, degrades to goitrin and prevents the thyroid hormones from being iodinated to triiodothyronine and thyroxine and finally results to thyroid hormones inactivation. If glucosinolate levels are high for prolonged time, impaired growth and goiter can develop [116]. This substantially reduces suitability of glucisinolate–reach rapeseed meal as a protein supplement [117]. Myrosinase is stored largely in the form of myrosin grains in the vacuoles of particular idioblasts called myrosin cells but has also been reported in protein bodies or vacuoles, and as cytosolic enzymes that tend to bind to membranes (Figure 5) [118,119].

Myrosinase from Sinapis alba exists as a dimer with subunits of 60–70 kDa each folded into a (b/a)8-barrel structure linked by a zinc atom and heavily glycosylated [120]. Enzyme structure is stabilized by salt bridges, disulfide bridges, hydrogen bonding, and glycosylation and activated by ascorbic acid. In contrast to many other β-glucosidases with catalytic glutamate residues at their active sites, in the case of myrosinase, both these residues are replaced by a single glutamine residue with ascorbate as cofactor acting as base catalyst [121,122].

Further degradation of hiohydroximate-O-sulfate aglucones is not dependent on myrosinase and proceeds with a formation of a mixture of products, their composition under physiological conditions is determined by the presence of supplementary proteins, known as specifier proteins, which have influence on enzymatic reactions but did not show enzymatic activity by themselves [123,124,125]. Without specifier proteins, the aglucone undergoes spontaneous Lossen rearrangement yielding isothiocyanates [126]. However, when specifier proteins are present, alternative hydrolysis products such as simple nitriles, epithionitriles, and organic thiocyanates are formed at the expense of isothiocyanates. Mechanism of specifier proteins interactions determining the composition of products formed by degradation of allyl glucosinolate (R = allyl) catalyzed by enzymes was thoroughly studied in Thlaspi arvense as a representative of other members of *Brassica* [127]. Specifier proteins are classified into epithiospecifier proteins, thiocyanate forming proteins, and nitrile-specifier proteins. Each of them can exist as different subtypes in different *Brassica species* and further differentiation of substrate specificity was observed even within specifier proteins produced in different organs such as roots or leaves [128].

### 4.2. Erucic Acid

Erucic acid is an unsaturated fatty acid with the configuration C22:1 n-9, which is known to cause heart lesions in experimental animals [129]. Its contents in recently introduced cultivars were reduced below harmful levels and therefore it is not considered a serious obstacle in the application of rapeseed products [23].

### 4.3. Sinapine and Other Polyphenolic Derivatives

**Figure 6:**
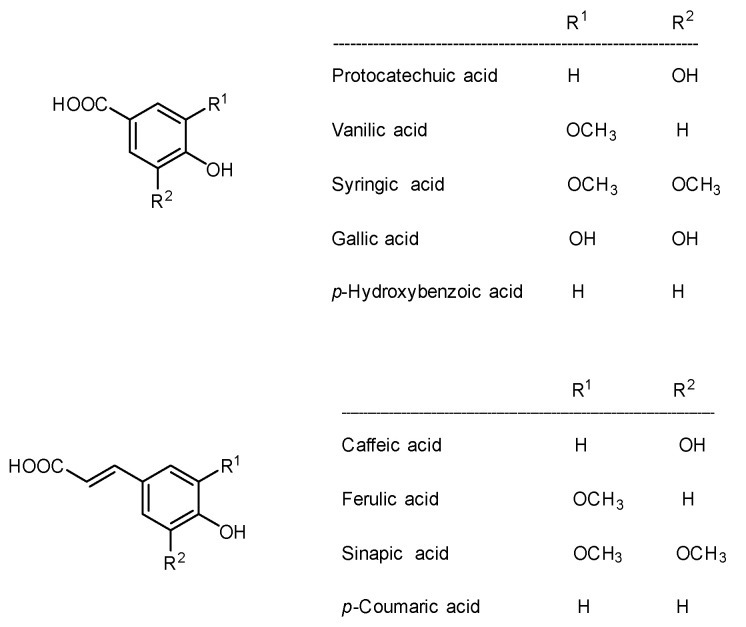
Structures of phenolic acids identified in rapeseed.

Tannins are polyphenolic biomolecules produced by plants that bind to and precipitate biomolecules including proteins, amino acids and alkaloids. Being widely distributed protects plants from predators and participate in regulating plant growth. Tannins are characterized by extensive structural heterogeneity with molecular weight ranging between 500 and 20,000 Da [40,130]. Several free, esterified, and insoluble phenolic acids were identified in the defatted rapeseed meals. The overall tannin content was reported to vary between 0.2% and 3.0% in the defatted rapeseed meal depending on the different cultivars, climatic effects, the analysis method, and the maturation stage [131]. The composition of the free phenolic fraction of tannin was determined by application of ultra-performance liquid chromatography (UPLC) system (Figure 6). Sinapic, p-coumaric, syringic, gallic, caffeic, and ferulic acids were identified as the main components of the fraction [132,133].

**Figure 7:**
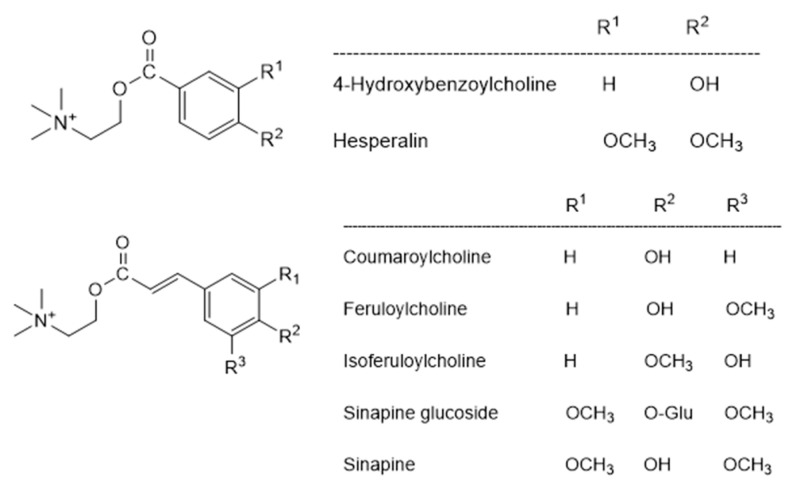
Choline esters of phenolic acids identified as components of rapeseed.

The most abundant was sinapic acid, constituting 71.7–94.5% (over 70%) of the total free phenolic acids (Figure 7). Esterified phenolic compounds were also identified and after being subjected to alkaline hydrolysis, the composition of phenolic acids was determined by UPLC.

In the fraction of esterified phenolic acids, sinapine, sinapoyl glucoside, and disinapoyl gentiobiose were identified with sinapic acid, constituting 98.3% to 99.6% of the total esterified phenolic acids. Sinapine is a choline ester of sinapic acid. It is the most abundant polyphenolic derivative present in fresh seeds of *B. napus* in a concentration 10–20 g/kg, sufficient to generate an unpleasant, bitter taste and astringency. The content of sinapine, as well as other polyphenolic derivatives, is steadily increased between the final stage of the green seeds and the ripening of the seeds [131].

Sinapine degrades to trimethylamine in dealkylation process. When subsequent enzymatic oxidation path of trimethylamine-to-trimethylamine oxide is damaged, the amine is accumulate. The impairment of sinapine metabolism can be result of inhibiting the activity of trimethylamine oxidase by the tannins or other polyphenols [131]. On the other hand, sinapine, at the concentration as present in the rapeseed extract exhibits, strong antioxidant properties, and has the ability to protect DNA from damage caused by the presence of a radical inducer and inhibits acetylcholinesterase activity by 85%, reducing the symptoms of Alzheimer’s disease and Myasthenia gravis disease [134,135,136,137,138]. Insoluble-bound phenolic acids ranged between 0.11% and –0.27% were determined by analyzing the residue obtained after extraction of free and esterified phenolic acids. Sinapic, protocatechuic (gentisic), p-coumaric, syringic, vanillic, gallic, caffeic, ferulic, salicylic, cinnamic, and 4-hydroxybenzoic acids were identified in the fraction. The main component was sinapic acid, constituting 83.4% to 96.2% [130,132]. When pressing the oil from rapeseed, most of the phenolic compounds remain intact in the meal. Further processing of rapeseed meals can strongly modify the sinapine content [139].

The antinutritional properties of tannins depend upon their chemical structure and dosage. At low dosages, tannins act as a potent antioxidant [140,141]. More than 95% of these compounds can be extracted from the canola seed cake using diluted ethanol. An extract rich in antioxidant compounds is a natural source of antioxidants [142]. In the European Union, the addiction of tannins as food flavorings (EC No 1334/2008, EU Regulation No. 872/12) is accepted, while the International Organization of Vine and Wine (OIV) restricts their application for protein binding and precipitation in must and wine.

### 4.4. Fibers

Non-starch polysaccharides, or more simply called fiber, have been most recently defined by the *Codex Alimentarius Committee of the Food and Agriculture Organization of the United Nations* (2010) “as those carbohydrate polymers with ten or more monomeric units which are not hydrolyzed by the endogenous enzymes nor absorbed in the small intestine.” The process of determining neutral detergent fiber content involves a neutral detergent that dissolves plant pectins, proteins, sugars and lipids. Insoluble remains are the fibrous parts such as cellulose, lignin and hemicellulose. Thus, determination of neutral detergent fiber is an important measurement used in evaluation of forage quality and is negatively correlated with energy concentration [143]. In order to determine individual components of neutral detergent fiber necessary for the evaluation digestibility of carbohydrate fraction, further analytical procedures are necessary. The residue obtained by treatment of plant material with dilute acid followed by extraction with solvent to remove starch, pectin, hemicellulose, fats, oils, protein, free sugars, and soluble minerals is called acid detergent fiber. Acid detergent fiber contains cellulose, lignin, and insoluble minerals (mainly silica) but not hemicellulose. Both methods, with some improvements, are still in use today for measuring contents of insoluble but dietary valuable fibers [144,145]. The limited digestibility of fibers influences the microbial population in the gastrointestinal tract and is found important in health and nutrient digestion. The nutritive value of undigestable fibers in the food is lost for the host but may promote colonization of digestive tract with pathogenic microorganisms [146,147].

### 4.5. Phytic Acid

**Figure 8:**
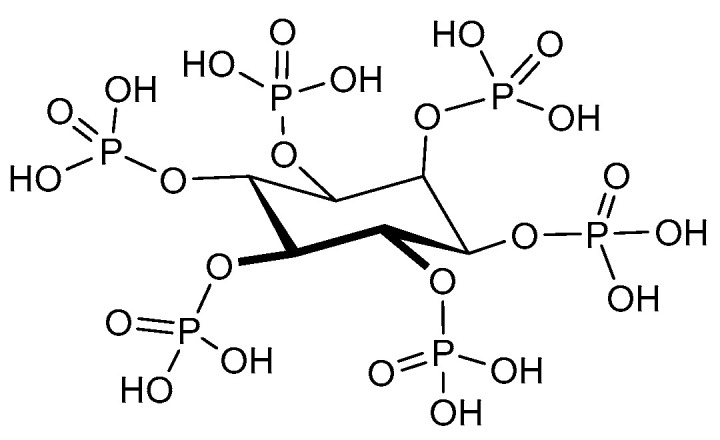
Structure of *myo*-inositol (1,2,3,4,5,6)-hexakisphosphate (phytic acid).

There are known numerous esters of inositol with phosphonic acid known as phytates. In plants, the most abundant is phytic acid formed by (1,2,3,4,5,6)-hexaphosphorylation of *myo*-inositol (Figure 8).

Phytic acid was identified in protein storage vacuoles in the aleurone cell layer but it is also present in the other tissues. Its primary function in seeds is as an energy source for the germinating seeds and mineral reserve of K, Mg, Ca, Mn, Zn cations stored in the form of salts with phosphate residues. Additionally, phytates show antioxidative ability by binding ferric irons, inactivation Fenton reaction and thereby preventing formation of hydroxyl radical [148,149]. Phytic acid also functions outside the plant kingdom and it was found in the brain and kidneys of rats [150,151]. In the case of a high phytic acid diet, the strong chelating effects of six phosphate group leads to low bioavailability of the minerals and can direct to their deficiencies and malnutrition. Moreover, phosphorous in the form of phytic acid is unavailable as a nutritional factor until hydrolysis of phosphates bond [152]. If not degraded, phytic acid phosphates are excreted and spread as manure into the soil, increasing the eutrophication of water, stimulating cyanobacterial blooms and death of several aquatic animals [153]. Therefore, degradation of phytic acid, if it is present in food, and the release of phosphorous and minerals have great interest to human and animal nutrition as well as preventing environmental pollution. Several groups of enzymes (phytases) were identified which are able stereo specifically hydrolyze phosphate from phytic acid with optimum activity depending on pH. The most tolerant on non-optimal reaction conditions and most interesting for industrial applications were phytases produced by microorganisms [154]. Recently, rapeseed mutants with significantly lowered phytic acid were obtained by gene editing [155].

## 5. Application of Microorganisms for Valorization of Rapeseed Meal and Cake

The problems arising from the generation of a large amount of waste material and depletion of fossil fuels deposits belong to challenges needed fast resolving in the present world. Conversion of waste into value-added products, including biofuels, is an economically viable and eco-friendly strategy to tackle simultaneously both issues [156,157]. It is estimated that globally 181.5 billion tons of lignocellulosic biomass are produced in a year [158,159]. Rapeseed straw (the solid residue obtained after harvesting rapeseed) is rich in cellulose and hemicellulose [160]. The cellulose content of rapeseed straw can range between 40 and 50%, with 15–25% hemicellulose and 15–20% lignin [161,162]. Currently, utilized are 7 billion tons coming from the forest, agriculture, and grass, while 1.2 billion tons are from agricultural residues [163,164]. The human annual consumption of biomass materials stands at nearly 72 billion tons, and by 2030 it is excepted to reach 100 billion tons [165,166]. This includes rapeseed meal produced approximately in the scale 40 megatons in 2019. The developed techniques and technologies of biomass valorization can be categorized into physical, chemical, physicochemical and biological categories [167,168]. Biological treatments are generally performed by growing microorganisms directly on the west materials or by the use of enzyme cocktails. Within this area, the progress made from ancient times is enormous [169,170].

An ample example is microbial fuel cell technology employing anaerobic microorganisms, which convert biodegradable substances into simpler substances and produce bioelectricity. Microbial fuel cells are different from chemical fuel cells as they depend on microorganisms as biocatalysts for the oxidation of organic-rich feedstocks to electrons, protons, and other byproducts. It is estimated that produced bioelectricity can reach 2–260 kWh/ton of waste material [171]. Another example is the development of biological pretreatment of biomass removing inhibitors such as furfurals, phenols and others and enabling application of classic technology for biomass conversion into valuable products [172,173,174]. Conversion of rapeseed agro-residues and residual material of rapeseed oil isolation to value-added products by applying microbiota is the economically viable and eco-friendly strategy to tackle this issue [175,176,177].

### 5.1. Substrate for Enzyme Production

The wide-ranging usefulness of enzymes in medicine, biochemistry, chemistry and many other areas is indisputable. Amylases, proteases, lipases, pullulanases, cellulases, chitinases, xylanases, pectinases, isomerases, esterases, dehydrogenases, and others have great potential applications for biotechnology, such as in agricultural, chemical, biomedical, and biotechnological processes. Microbiota are excellent sources of them owing to their broad biochemical diversity and their suitability for genetic manipulations. The microbial enzymes are not subjected to any of the production and supply limitations, offering simple upscaling procedures [4].

There are two trends visible to obtain microbial enzymes. In the classic approach, the preferable are procedures yield a homogeneous enzyme as the final product. According to the second approach, acceptable product consists a mixture, intensively fortified with the one, expected enzyme, accompanied by other tolerable components. This old fermentation technique was practiced since ancient times in many modifications. Microbial enzyme production follows two main strategies: isolating highly purified enzymes or producing enzyme-rich crude extracts, the latter being a long-standing fermentation practice. These enzymes can be generated via liquid-based submerged fermentation or through solid-state fermentation (SSF), which utilizes minimal water and is increasingly favored for its efficiency, higher product yields, and reduced operational costs [178,179,180]. The success of enzyme production hinges on optimizing a multitude of interacting factors, including the microbial strain, nutrient media composition, inoculum preparation, and physical parameters like pH and temperature, often guided by mathematical models [181,182].

Enzyme production strategies generally aim to obtain either high-purity enzymes or crude enzyme extracts. High-purity enzymes are isolated through multistep purification processes and are essential where substrate specificity, regulatory compliance, or reaction predictability is critical—such as in medical or pharmaceutical applications. Crude enzyme extracts, in contrast, are less refined mixtures of active enzymes and accompanying biomolecules that can be recovered directly from fermentation media. These are frequently used in agro-industrial or feed applications due to their lower cost, ease of production, and potential for synergistic activity. The choice between these approaches depends on the target application, required purity level, and economic considerations.

The preferences to the production of the given enzyme depend on the microorganism strain, culture media and their supplementation with additional components (if any), preparation of inoculum, fermentation system and applied conditions including pH, temperature, and many others. The optimization of this multidimensional relations between parameters of the fermentation process is possible by appropriate mathematical functions [183]. In the rapeseed cake, the presence of antinutrients is limiting the growth of several microorganisms, especially bacteria, however, this restraint can be evaluated as beneficial for growing species tolerating such components. As the most tolerant towards antinutrients were found fungi, especially isolated from decayed rapeseed-waste material.

The bioconversion of rapeseed meals by bacteria and yeast is often limited by the lack of extracellular hydrolytic enzymes in these microorganisms. When valorizing rapeseed cake, a key challenge arises from its antinutrient content, which can inhibit many microorganisms, particularly bacteria. This same characteristic, however, can create a selective advantage for more tolerant species, notably fungi, especially those naturally adapted to rapeseed environments. Furthermore, many bacteria and yeasts lack the necessary extracellular hydrolytic enzymes to efficiently break down complex rapeseed meal components. To overcome this, a synergistic approach often involves an initial fermentation step with hydrolytic enzyme-producing fungi. These fungi effectively pre-digest the meal, releasing simpler, bioavailable nutrients such as amino acids, phosphorus, and glucose. This enriched medium subsequently supports more robust growth and product formation by other target microorganisms, transforming the rapeseed meal into a versatile feedstock for broader biotechnological applications [184,185,186,187].

#### 5.1.1. Lipase

Lipases (triacyleglycerol acylhydrolase EC 3.1.1.3) are a group of hydrolytic enzymes involved in the hydrolysis and synthesis of triacylglycerides in the oil–water interface. Lipases as biocatalysts found a large number of applications in food, dairy, detergent, pharmaceutical, biotechnology, oleochemical, and many other industries. The enzymes are used in the oleochemical industry for the production of fatty acids from oils through hydrolysis. Fatty acids found wide applications as starting materials used for manufacturing surfactants and biofuels, in the food industry and in many biomedical applications. Biocatalytic efficiency of lipases is also employed for esterification and transesterification reactions useful in the synthesis of flavoring components, enantioselective preparation of optically active building blocks and many other advanced products of organic synthesis. Their synthetic versatility and substrate/product preferences vary and depends on the strain and even the strain variant of microorganism producer [188,189].

For the production of lipase, Ganoderma lucidum grew on canola oil cake under solid-state fermentation at 30 °C. The maximum yield of enzyme was obtained at pH 4.5 using 60% moisture level, olive oil as inducer (2.0%) and 96 h incubation period. Under optimized conditions, solid-state fermentation of Ganoderma lucidum on canola oil cake yielded 4838 U/g of extracellular lipase in the culture supernatant as assayed spectrometrically using p-nitrophenyl palmitate as substrate [190]. The enzyme was isolated from the supernatant by fractional precipitation followed by ion exchange chromatography and gel filtration on Sephadex G-100 column with 2.26% recovery. A temperature of 30 °C and a pH 8.5 of were found to be optimal for the activity of isolated enzyme. Thermostability, kinetic Michaelis–Menten parameters and energy of activation for p-nitrophenyl palmitate hydrolysis were determined [191]. *Panicyllium species* were also found to be useful for the production of lipases. After screening several process parameters and 26 different filamentous fungi, *Penicillium camemberti* AM83 was found to be the most efficient producer of lipase and protease in solid-state fermentation on rapeseed cake [192]. Canola oilseed cake was found superior substrate for solid-state fermentation using *Penicillium notatum* as compared to linseed oil cake, cotton oilseed cake, rice bran and wheat bran. Under optimized conditions, the lipase activity of 5335 U/g dry substrate was obtained at pH 5 after 96 h of incubation period at 30° [193]. The superiority of canola meal as substrate for the production of lipase from different agro-industrial wastes such as cotton-oilseed cake, linseed-oil cake, sesame-oilseed cake, rice bran and wheat bran were also observed in solid-state fermentation using *Pleurotus ostreatus* IBL-02 [194], *Penicillium fellutanum* [195], *Aspergillus melleus* [196] and *Aspergillus oryzae* cultivated in an instrumented lab-scale bioreactor under solid-state fermentation with canola cake as a sole carbon source [197]. Lipase preferentially transforming triglycerides of medium and long-chain fatty acids was produced by *Aspergillus niger* [198].

The further progress in lipase production from canola cake was achieved by using *Yarrowia lipolytica*, the strain generally recognized as safe by the Food and Drug Administration (FDA, USA). This broadened the scope of the application of crude lipase extracts as versatile biocatalysts, once they presented significant hydrolytic activity under different conditions of temperature and pH. Cautiously optimized composition of carbon and nitrogen sources demonstrated the high biotechnological potential of canola cake feedstock for solid-state fermentation [199,200]. The potential of biocatalytic application of lipase crude extracts was reviewed recently [201].

#### 5.1.2. Phytase

Phytic acid, existing mostly in the form of salts with multivalent cations, is a component of mature seeds, supplying 85% of phosphorous during the germination of rapeseed [202,203]. However, inorganic phosphorous become available for metabolic processes only after hydrolysis of phytic acid. Phytases (*myo*-inositol hexakisphosphate 3-phosphohydrolase, EC 3.1.3.8. and *myo*-inositol hexakisphosphate 6-phosphorylase, EC 3.1.3.26.) are family of histidine acid phosphatases that catalyze the hydrolysis of phosphate ester bond in phytic acid affording finally inorganic phosphate and *myo*-inositol [204,205]. Due to deficiency of this enzyme in gastrointestinal tract of monogastric species, phytic acid is undigestable, which limits the availability of mineral nutrients and phosphorous by non-ruminants [206,207,208,209,210,211] and deteriorate the activity of several enzymes such as amylase, trypsin, tyrosinase and pepsin [183]. Moreover, undegraded phytates in manure are hydrolyzed in soil causing serious phosphorus pollution advancing eutrophication of surface waters [212]. Both above mentioned problems can be resolved by supplementation of phytate reach foodstuffs with phytase.

In the search for the phytase producing filamentous fungi, three *Mucor* and eight *Rhizopus* strains were grown on various natural substrates by solid-state fermentation. For the rapeseed cake, a relatively good yield of phytase was obtained in fermentation using *Mucor* racemosus NRRL 1994, *Rhizopus* oligosporus NRRL 5905, and *Rhizopus* oryzae NRRL 1891. However, for *Mucor* racemosus NRRL 1994 the higher yield (26 IU/g dry matter phytase activity) were obtained using coconut oil cake supplemented with glucose, casein and ammonium sulfate then rapeseed cake [213]. One of the most efficient phytase producers are various *Aspergillus* strains. Satisfying results of phytase production in canola meal were obtained using *Aspergillus carbonarius* [214,215]. Canola meal was also found useful for phytase production by thermotolerant *Aspergillus niger*, although using cowpea meal as the growth medium for the highest enzyme activity (108 U g^−1^ dry moldy bran) obtained [216]. Optimization of solid-state fermentation conditions was found essential for improvement enzymatic procedures applied for the treatment of rapeseed meal with microorganisms.

Mathematical correlations of phytase activity with parameters of solid-state fermentation of canola meal using *Aspergillus ficuum* NRRL 3135 were determined. The progress of the process was monitored by determination of enzyme activity and amount of enzyme in the crude extracts from canola meal culture during the fermentation. According to the studies, optimum moisture content of the solid medium for was 64%, inoculum age was between 2 and 5 days and inoculum homogenization was recommended [183].

In the process of optimization of the production of phytase, besides classic studies on the effects of moisture content, supplementation with glucose, phosphate, and surfactants, gamma irradiation was regarded as additional important parameter determining efficiency of the solid-state fermentation of rapeseed meal using *Aspergillus niger* A-98. It has been revealed that gamma irradiation dose 1 kGy increase 1.35 times higher secretion of phytase in small scale experiments. Irradiation dose below 0.2 kGy or above 1.25 kGy not effected phytase production [217]. The mechanism of activation of phytase secretion was not proposed, although the stimulating mutagenic effect of irradiation on the production of other enzyme by *Aspergillus niger* was reported [218]. In neither case, however, the stimulating effect of mutagens was confirmed by passing microorganism strains, which could reject assumption that stress conditions were decisive for increase enzyme production.

Recently, in order to increase the productivity of enzyme microorganisms used in the fermentation process, they were subjected to the intentional genetic modification. To improve the phytase-based phosphorus recovery process, the codon-optimized gene of *E. coli* AppA phytase (Ec phy) was cloned in the pBSYA1S1Z vector and overexpressed in *Pichia pastoris* BSYBG11, which is well established for the rapid and cost-effective expression of recombinant proteins [219,220].

#### 5.1.3. Alkaline Protease

Proteases consist a large group of hydrolytic enzymes able to cleave the peptide bond located at different, yet precisely defined positions, of the protein chain. Proteolytic enzymes are present in all living organisms, yet microbiota is accepted to be the most versatile industrial-scale producers [133,221]. The activity profiles of proteases are strongly diversified. Alkaline proteases are produced by alkalophilic microorganisms. This enzymes found extensive application in several industrial sectors, especially in detergent manufacturing and leather industries [222,223].

The screening of versatility of different agro-industrial waste materials for the production of alkaline protease by submerged and solid-state fermentation using wild as well as the mutant *Bacillus subtilis* IH-72EMS8 strain showed moderate usefulness rapeseed meal as culture medium, although its potential has been not fully recognized in the studies [224].

Superiority of rapeseed meal culture medium were found in the manufacturing rennin-like acid protease promoting milk clotting and therefore vital for manufacturing dairy products as substitute of rennet extract. The enzyme is produced by strains of *Mucor miehei*, *Mucor pusillus Penicillium*, *Aspergillus*, *Trametes sanguinea* and others. Selected from eight mold cultures belonging to the genus *Mucor* strain identified as *Mucor pusillus* IHS6 produced 75 U/mL of the enzyme growing on rapeseed meal [225,226].

#### 5.1.4. Nattokinase

**Figure 9:**
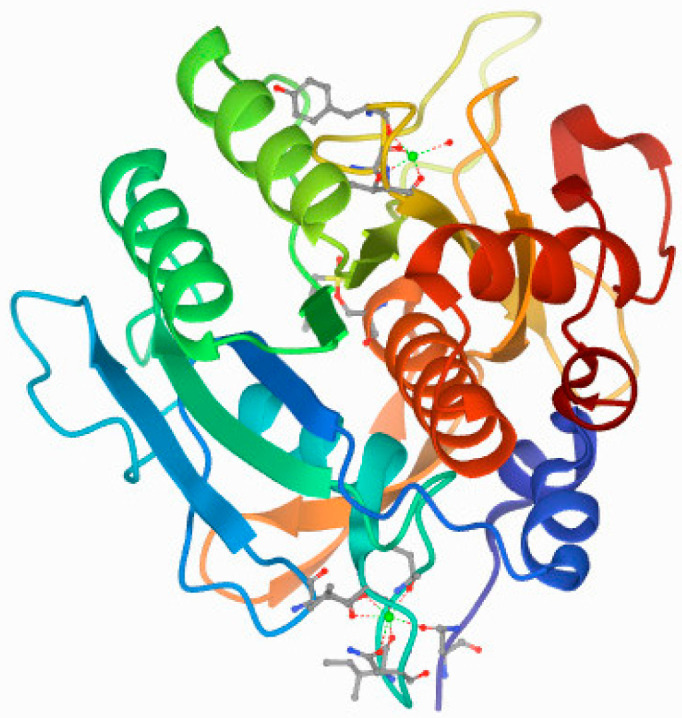
Crystal structure of nattokinase produced by *Bacillus subtilis* natto.

Fermentation of boiled soybeans with a special type of bacteria named *Bacillus subtilis* natto produce Japanese food known as natto. The active component of natto is a strongly fibrinolytic enzyme, nattokinase. The enzyme is a polypeptide composed of 275 amino acids with the structure determined by crystallographic studies (Figure 9) [227,228].

Nattokinase is evaluated as highly beneficial for treatment cardiovascular diseases, reducing blood pressure, high cholesterol, and stroke risk, but there is no good scientific evidence to support any of these uses. By UV radiation induced mutations of *Bacillus natto* Z1 strains followed by repetitive screening and testing genetic stability of resulted mutants, the new strain named *Bacillus natto* U49 was obtained, which was able to produce nattokinase effectively utilizing rapeseed meal as basic nitrogen source [229].

### 5.2. Enzymes Degrading Polysaccharides

The enzymes performing hydrolysis of various glycosides and oligosaccharides are vital for degradation of plant cell walls and transformation of plant matter into usable nutrients and form many other applications in industrial biotechnology. The main components of plant cell walls are cellulose, hemicellulose and lignins. Pectins are polysaccharides rich in uronic acid. Due to the diversified structure and chemical composition of pectin among plants, their degradation into sugars and uronic acid requires the palette of pectinolytic enzymes. In the search for efficient manufacturing of pectinolytic enzymes by the Penicillium oxalicum strain, various agro-industrial residual materials were tested. In submerged and solid-state fermentation, rapeseed cake was found useful as substrate, although less productive then wheat bran [230,231].

#### Xylanase (Beta Glucanase)

Xylanase (EC 3.2.1.8) degrade hemicellulose by hydrolysis of xylan into xylose. Xylanases are produced by fungi, bacteria, yeast, marine algae, protozoans, snails, crustaceans, insect, seeds, filamentous fungi, etc., but not by mammals [232]. It is used in large amounts in the pulp and paper industry and also as food additives often in combination with other polysaccharide hydrolases [233].

In the search for substrate useful for the production of xylanase *Trichoderrna reesei,* it was found that canola meal supervene Solka-floc, xylan or glucose. The maximum xylanase productivity using canola meal was 210 IU/mL in 9–12 days. The obtained isolate also contained acetyl-xylan esterase, cellulase, xylosidase and effectively hydrolyzed a variety of waste polysaccharide into fermentable sugars [234].

The stimulating effect of mustard cake constituent in fermentation medium, inducing the xylanase production by *Trichoderma koeningi* and β-glucosidase by *Trichoderma viride* was also observed in the independent studies [235,236]. The fermentation with *Trichoderma* species proceeded effectively using several biowaste materials and under broad range of fermentation conditions. This open the promising possibilities for the conversion of agro-industrial residues cellulosic material [237].

### 5.3. Multienzyme Producers

Every microorganism produces a palette of enzymes, but few excrete them in amounts sufficient for economically justified isolation and separation. In many cases, the composition of different enzymes offers significant advantages over individual enzyme. Another important benefit of this practice is relatively easy modification of quantitative relation between components of the mixture, even by simple alternation of fermentation conditions when using the single microbiota strain [238].

In the search for species producing thermophilic protease, a range of *Actinomycete* were tested. The highest proteolytic activity was obtained when *Streptomyces thermovulgaris* was grown on rapeseed meal-derived media [239]. Analysis reveals that *Streptomyes thermovulgaris* secreted two types of serine protease, azocaseinase and metallo-type protease. Growing the thermophilic *Streptomyces* on yeast extract medium produced a different range of protease enzymes [240].

Solid-state fermentation cold-pressed canola cake by the *Aspergillus ficuum* DSM 932 strain produced phytase, cellulase and xylanase. For the production of phytase, a pH of 6 and a temperature of 60 °C were optimal. The production of cellulase was optimal at pH 7 and xylanase at pH 5.4 at an optimal temperature of 45 °C [241]. It is interesting to note that the enzymatic profile produced by the same *Aspergillus ficuum* DSM 932 strain can be dramatically altered by modification of growing media. Substitution of rapeseed cake by cranberry pomace deteriorated the production of phytase, cellulase and xylanase but strongly favored protease [242].

Cellulase (EC 3.2.1.4) was obtained as a side-product of oxalic acid manufacturing by rapeseed fermentation using *Aspergillus niger* strains S, C-12, and W-78-B. Under this process conditions xylanase (EC 3.2.1.8) was obtained only in presence of *Aspergillus niger* strains S [243].

Canola meal was found superior substrate for production xylanase by *Trichoderma reesi.* Maximum xylanase productivity reached 210 IU/mL in 9–12 days. The obtained enzyme system was enriched with acetylxylan esterase, cellulase, and xylosidase and found very useful for transformation of corn cobs, corn, wheat bran, straw, and larchwood xylan to fermentable sugars [234,238]. The most efficient and most universal pathway to the preferred composition of enzymes was, however, the co-fermentation of selected microorganisms consortia.

### 5.4. Microbiota Supported Organic Synthesis

The most efficient and most universal pathway to the preferred composition of enzymes was, however, the co-fermentation of selected microorganisms consortia. Microbiota cells are complex chemical factories where all available resources, including bio-organic waste materials, can be transformed into palette of valuable chemicals, under ambient conditions. Synthetic biology, known also as precision fermentation, has been developed into technologies used in large-scale production biopigments, biopolymers, biosurfactants, acrylic and lactic acid, biodiesel, natural sweeteners such as sorbitol and xylitol and many other products [4,244].

There is also rapidly developing application of microbiota cells as tools for precisely defined chemical transformation substituting regular chemical reagents and processes.

#### 5.4.1. Whisky Lactone

**Scheme 3:**
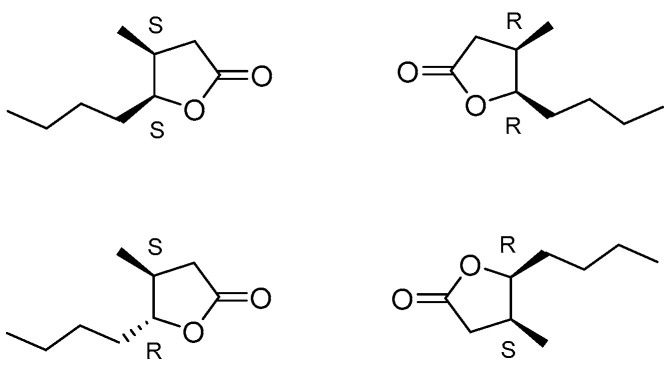
Four stereoisomers of 5-butyl-4-methyloxolan-2-one.

Each of the four stereoisomers of 5-butyl-4-methyloxolan-2-one found in nature provides a different fragrance [245]. Stereoisomer (4S,5S) and (4S,5R) of 5-butyl-4-methyloxolan-2-one, known as whisky lactone (oak lactone or quercus lactone) (Scheme 3) are important ingredient in the aroma of whiskey, and other alcoholic beverages [246]. The mixture of stereoisomers is readily available by synthesis.

Access to optically pure forms of both diastereomers 5-butyl-4-methyloxolan-2-one was achieved in two steps. In the first one, separation of both diastereomers yielded two mixtures of enantiomeric forms. For resolution of enantiomers by solid-state fermentation of rapeseed cake, several strains of filamentous fungi were tested [247]. After optimization of process condition, *Fusarium oxysporum* AM13 resolved a both mixtures of *trans*- and *cis*-5-butyl-4-methyloxolan-2-one. *Trans*-(+)-(4S,5R) and *cis*-(+)-(4R,5R) enantiomers were obtained with enantiomeric excess exceeding 99% and 98%, respectively [248,249].

#### 5.4.2. Surfactin Analogues

Lipopeptides are a strongly diversified group of biosurfactants formed by a fatty acid (between C12 and C18) linked to a linear or cyclic peptides (depsipeptides) composed from 4 to 12 amino acid residues [250]. They are synthesized by nonribosomal peptide synthetases, which lead to a remarkable heterogeneity with regard to the configuration, type and sequence of amino acid residues, the length, functionality and branching of the fatty acid chain [251].

Biosurfactants are mainly produced by Bacillus and Pseudomonas genera and their potent antimicrobial and mostly antifungal activities may differ from one compound to another depending on the structural details [252]. The best known cyclic lipopeptides are surfactins, iturins, fengycins, lichenysins, viscosins, amphisins and putisolvins [253,254].

**Scheme 4:**
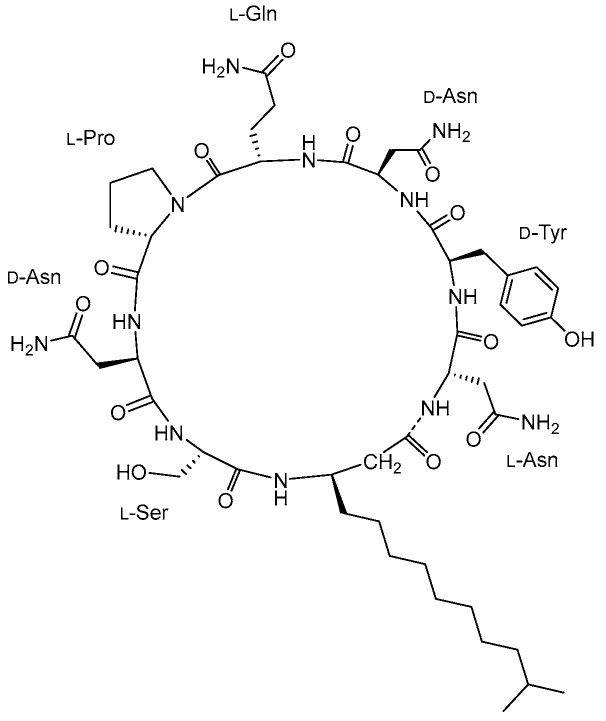
Structure of iturin A.

Isolated from soil and identified as *B. subtilis*, strain 3–10 produced iturin A (Scheme 4) and poly-γ-glutamic acid under solid-state fermentation on rapeseed meal.

After optimization of the fermentation procedure, productivity reached: iturin A 5.3 g/kg of dry substrate and poly-γ-glutamic acid 51.3 g/kg, respectively [255]. Further improvement of fermentation procedures involving Bacillus subtilis for the production of iturin A were reported [256,257].

The effects of antinutrient components of rapeseed meal on submerged fermentation of the Bacillus amyloliquefaciens CX-20 strain was thoroughly studied. The protein pool and mineral supported, whereas fiber reduced, iturin formation. The fermentation broth remaining after lipopeptide isolation was found active as natural, potent plant-protective agent and additionally biofertilizer, promoting the growth of Brassica napus [258]. The pre-treatment of rapeseed meal with enzymes or fungi further improved direct bio-utilization of this substrate in fermentation with the Bacillus amyloliquefaciens CX-20 strain [259,260].

The process of lipopeptides synthesis on nonribosomal peptide synthetases is not strictly conservative and enable intensive modification of lipopeptide structure by fermentation conditions and diversity of the microbial strain.

**Scheme 5:**
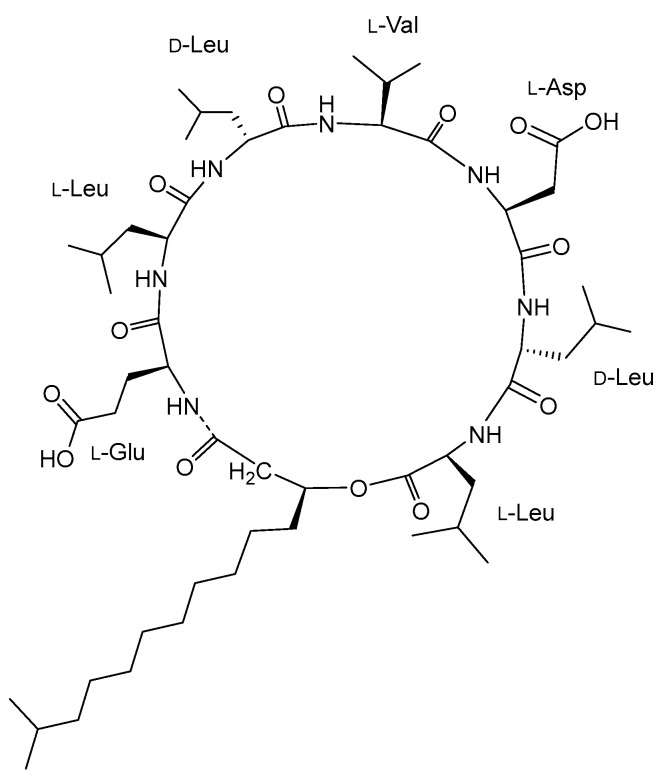
Structure of surfactin.

In the synthesis of surfactin (Scheme 5) analogues by *Bacillus subtilis* strains KB1 and #309 growing on rapeseed cake, the crucial factor determining the structure of preferred surfactin analogue was oxygen supply. A lower oxygen amount decreased the share of C15 analogs while it increased the share of C12 analogues [261].

#### 5.4.3. 1,3-Propandiol

Due to the rapidly increased scale of biodiesel production from rapeseed oil, up to 45 billion liters in 2020, large amounts of glycerol isolated as the side-product entered the world market exceeding the demand for its application (glycerol) as a chemical. This stimulated the search for alternative pathways of it conversion to higher-value products. 1,3-Propanediol is one of the products that could be obtained by the transformation of crude glycerol. 1,3-propanediol is used as an important monomer in the manufacture of biodegradable polyesters, cosmetics, cleaning agents, lubricants, engine coolants, heat transfer fluids, de-icing fluids, water-based inks, etc. [262,263].

Glycerol can be converted to 1,3-propandiol by species belonging to Clostridium, Lactobacillus and Enterobacteriaceae. After screening 4000 strains of the Enterobacteriaceae family, it was found that 28% of them could synthesize 1,3-propandiol from glycerol. The best producers were Klebsiella pneumoniae, Klebsiella oxytoca, Citrobacter freundii and Hafnia alvei. After optimization of fermentation conditions, in bioreactor scale, Citrobacter freundii AD119 gave 23 g/L of 1,3-propandiol [264,265]. In independent studies, from nearly 4000 Enterobacteriaceae strains, Clostridium butyricum VPI 1718 was selected as the most productive in transformation of glycerol into 1,3-propanediol when growing on rapeseed meal hydrolysate [266,267].

#### 5.4.4. Omega-3 Docosahexaenoic Acid

**Figure 10:**
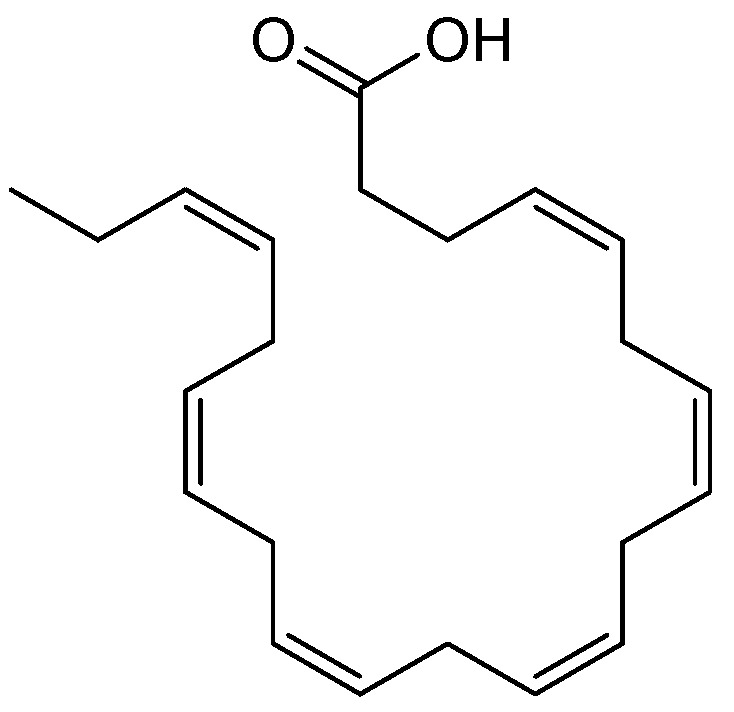
All-cis-docosa-4,7,10,13,16,19-hexaenoic acid, (22:6(n−3) acid, cervonic acid).

All-cis-docosa-4,7,10,13,16,19-hexaenoic acid (Figure 10) is the most abundant exogenic omega-3 fatty acid in the brain and retina. It is an important structural components in cell membrane phospholipid bilayers, nodulate anti-inflammatory lipid mediators inhibiting the production of interleukin-6 and interleukin-8 in human endothelial cells. It also interacts with the G protein-coupled receptor, affects transmembrane transport, promote apoptosis, neuronal differentiation and ion channel activity [268,269,270]. The beneficial effects of docosahexaenoic acid supplementation of food products as well as its application as pharmaceutical components substantially increases demand for this product.

The condition in which the microalgae have grown highly influences the lipid formation. Stressing environmental changes stimulate production and accumulation of microalgal lipids [271,272,273]. Several other classic fermentation procedures leading to complex products were improved by application of rapeseed meal as substrate or as source of nitrogen. Common microbial fermentation processes (e.g., for lactic acid, ethanol, enzymes, citric acid, SCP) were enhanced or made more cost-effective by using rapeseed meal—either as the main nutrient source or as an additive to support microbial growth and productivity.

**Scheme 6:**
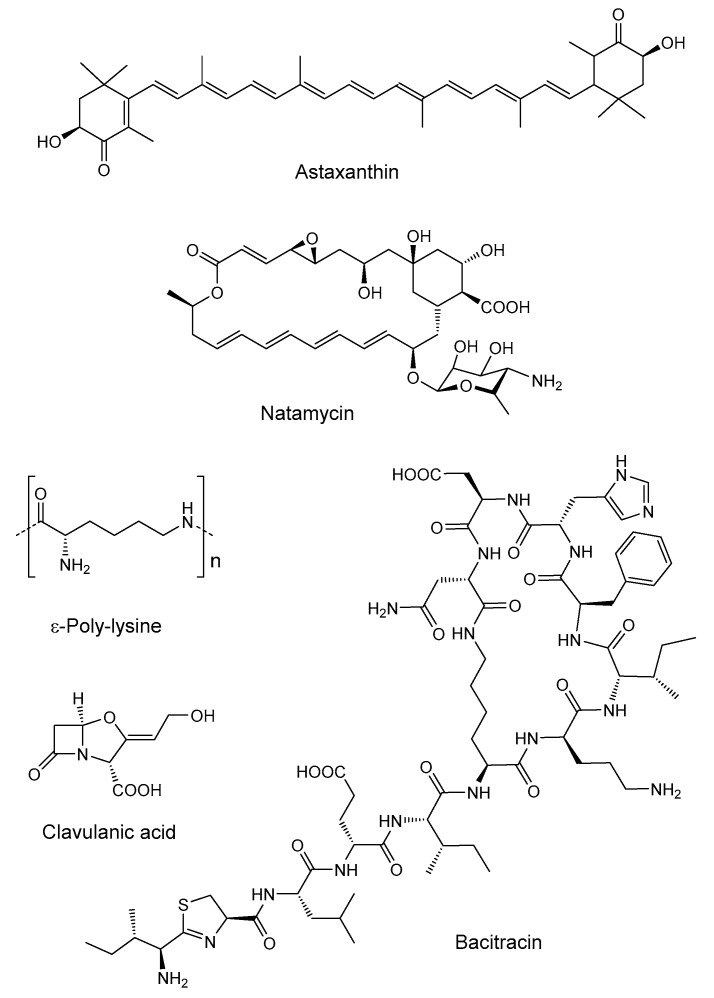
Structure of other products obtained by rapeseed meal fermentation.

This included clavulanic acid used to protect β-lactam antibiotics from degradation by β-lactamase [274], polypeptide antibiotic Bacitracin (Scheme 6) produced by *Bacillus licheniformis* DW2 [275], Natamycin (Scheme 6) also known as pimaricin an antifungal medication also used in the food industry as a preservative [276], ε-polylysine (Scheme 6) used commercially as a food preservative, antimicrobial agent and in drug delivery system [277].

The several less structurally defined products were obtained by microbial treatment of rapeseed meal. The aqueous extracts obtained after a solid-state cultivation of filamentous fungi *Aspergillus sojae* on rapeseed meal exhibited interesting immunomodulatory activities in immune cell models through IL-1β secretion of LPS-stimulated cells such as neutrophils, peripheral blood monocytes or macrophages [278], carbohydride polymers with probiotic prosperities which can even serve as alternatives to antibiotics, the polymers with moisturizing properties used in cosmetics, biosurfactants produced by bacteria and yeast with strong antimicrobial effects and many other products are available as components of post-fermentation broth obtained by treatment of rapeseed meal with microbial generally recognized as safe [244].

### 5.5. Neutralization of Antifeedant and Enhancing Nutritional Value of Rapeseed Meal by Treatment with Microrganizms

The utilization of crude rapeseed meal as a protein source is restricted due to toxicity of products formed by degradation of glucosinolates, the high content of undigestable crude fibers, the presence of unpalatable tannins which are prone to form insoluble complexes with proteins, thus limiting their digestibility, and the last but not least, occurrence of phytates reducing the minerals bioavailability [279]. However, favorable ratio of n-6/n-3 polyunsaturated fatty acids (PUFA) in residual oil, well-balanced essential amino acids composition, significant amount of minerals and a wide variety of vitamins strongly stimulated the efforts to eradicate the antifeedant from rapeseed meal [280]. Several strategies are presented to minimize the deleterious effects of this antinutrients components on rapeseed proteins bioavailability. This include technologies of protein extraction from rapeseed, removing antifeedant by solvent extraction, supplementation of food with enzymes [281], optimization of multiple physicochemical and biochemical parameters using response surface methodology and determination of their influence on rapeseed meal detoxification by Design-Expert 7.1.6 software [282] and others [283]. The progress achieved in this area allows transformation of rapeseed meal into rapeseed protein isolate (Isolexx) accepted by EU commission as the novel nutrient [284,285].

The simple approach based on high-temperature deactivation of rapeseed myrosinase is often unproductive, because glucosinolates can be decomposed to toxic products by analogously active enzymes produced by microorganisms of intestinal tract or soil. Even in the absence of the enzymes, glucosinolates can be degraded to the harmful compounds by inorganic catalytic systems.

Nonetheless, the high cost and limited availability of components necessary for preparation of appropriate palette of degrading enzymes can severely limit the scope of this solution. Thus, the alternative and very promising technology was developed base on in situ application of enzymes produced by selected microbiota strains. A promising solution involves using bacteria generally regarded as safe to biotransform rapeseed meal into more valuable nutrients. Vig and Walia found that fermentation of rapeseed meal by *Rhizopus oligosporus* reduced the concentration of glucosinolates, phytic acid and crude fiber by 43.1%, 42.4% and 25.5%, respectively [18,286,287].

The efficiency of the *Rhisopus* strain in the reduction in antifeedant components of rapeseed meal was further confirmed by independent studies [288]. Selected glucosinolate-degrading strains improved the quality of rapeseed meal by reducing the phytic acid and crude fiber by 28.3% and 66.2%, respectively [289]. Treatment of rapeseed meal with myrosinase producing fungi was found effective approach for decreasing glucosinolate content. Solid-state fermentation of rapeseed cake, supplemented with wheat bran, using *Aspergillus niger* degraded glucosinolates and upgraded nutritional value of the product. The fermentation increased the enzyme activity of endoglucanase, xylanase, acid protease and phytase. After optimization of the process conditions, in the mixture with 70% rapeseed cake, degradation of 76.84% glucosinolates was attained at 34 °C at 72 h. Under this conditions total amino acid contents in dry mas increased from 277.95 g/kg to 345.54 k/kg with improving essential amino acid content from 122.64 g/kg to 157.56 g/kg accompanied with substantial decreased amount of other antinutrients. The contents of none dietary fibers and phytic acid declined by 9.12 and 44.60%, respectively [290].

A substantial decrease in antinutrients was obtained by treatment of rapeseed meal with *Aspergillus niger* in a two-step procedure. In the first step, rapeseed meal fortified with 30% wheat bran was fermented at 30 °C for 48 h. After enzymatic hydrolysis at 45 °C for 24 h, in the second step, the content of trichloroacetic acid soluble protein fraction increased by 81.70% and glucosinolates reduced by 30.06%, respectively [291]. Screening 10 glucosinolates-degrading strains selected *Aspergillus* flavus as degrading up to 58% glucosinolates and *Aspergillus niger* reducing content of tannins and phytic acid and increasing the content of crud protein from 37% to 43% [292,293]. Moreover, there are observation of modification of the amino acid profile content after fermentation using *Aspergillus niger*. Increase in the content of histidine by 5.6% and arginine by 1.78% was accompanied by declined content of aspartic acid and glutamic acid. This strongly suggests that both acidic amino acids are consumed in process of both mentioned above basic amino acids [290,291].

Enhanced catalytic efficiency of myrosinase degrading enzyme isolated from *Aspergillus* sp. NR4617 was obtained by the mutagenesis of wild-type fungus after treatment with methanesulfonate and N-methyl-N’-nitro-N-nitrosoguanidine [294]. The mutant strains retained activity profile analogous to wild-type *Aspergillus* sp. NR4617E1 [295].

Even more promising results were obtained using composite strains for degradation of worthless components of rapeseed meal. Preliminary screening selected 67 strains containing 13 yeast, 41 bacteria and 13 molds exhibiting growth on the glucosinolate-containing culture medium. Single-strain fermentation resulted in the consumption of less than 70% glucosinolates, whereas mixed strain fermentation consumed up to 92% glucosinolates. Further studies revealed that co-fermentation of two molds (*Lichtheimia* sp. JN3C and *Aspergillus terreus*) with yeast additionally increase the protein content to 48.2%. Moreover, while in the 96 h fermented rapeseed meal when 3 microorganisms were cultured simultaneously with 13.2 g dry weight of product containing 38% proteins, 63.5 μmol/g glucosinolates, 14.2% lignocellulosic fibers, 5.3% phytic acid and 0.95% tannins, the total amount of isolated dry product was 11.7 g with amount of proteins increased to 48.4%, and the quantity of antinutrient components decreased to 1.3 μmol/g, 4.8%, 3.8%, 1.10%, respectively [289].

A study on aerobic and anaerobic, two state fermentation of plant protein raw material composed mainly from rapeseed meal, soybean meal, and cottonseed meal by *Aspergillus niger*, *Bacillus subitilis*, *Lactobacillus plantarum*, Candida tropicalis as the starting strains confirmed a substantial increase their nutritional value. The crude protein content increased to 45.47% within acid-soluble protein 159.2 mg/g accompanied by decrease in detoxification of glucosinolate and gossypol by 83.77% and 89.82%, respectively [291,294].

Fermentation of rapeseed meal with 1: 1 mixture of *Lactobacillus fermentum* and *Bacillus subtilis* under anaerobic conditions increased crude protein contents from 37.1% to 39.6%, within lysine contribution ranging from 1.54% to 2.68%. At the same time isothiocyanate antinutrients were reduced from 108.7 mmol/kg to 13.1 mmol/kg. The results suggest that rapeseed meal fermented with *Lactobacillus fermentum* and *Bacillus subtilis* is a promising alternative protein source and that it could be safely used replace up to 10% soybean meal in broiler diets [296].

Substantial improvements of the nutritional value, sensory properties and bioavailability were attained by fermentation of rapeseed meal with a consortium of *Bacillus licheniformis* (1.0813), Yeast (ACCC20060) and *Lactobacillus* (ACCC10637). The soluble protein, lactic acid and total amino acid content increased significantly after 3 days of fermentation, whereas the glucosinolate and neutral detergent fiber content decreased significantly. Moreover, most proteins were degraded to small peptides with molecular weights less than 9.5 kDa by microbial treatment [297].

Screening of 67 strains containing 13 yeast, 41 bacteria and 13 molds isolated from the decayed rapeseed meal selected myrosinase producing fungi, *Lichtheimia* sp. JN3C and *Aspergillus terreus* as most promising for degradation of antinutrients [291,298].

In Chinese literature, the benefits of using mixed strains for detoxification of rapeseed meal were documented in several other reports [299,300,301,302]. Although, there is also one report presenting the opposite declaration after comparing results of rapeseed meal fermentation by single and mixed strains of *Bacillus subtilis* W1-3, *Bacillus subtilis* 10160, fungi: *Pichia*, *Saccharomyces cerevisiae* and *Aspergillus niger*. The screening showed that optimal results of rapeseed meal detoxification and increase of nutrient components were obtained using *Bacillus subtilis* W1-3. In the single bacterium fermentation strain, the content of glucosinolates and phytic acid decreased by 62.14% and 31.58%, and nutrient substances such as peptides, crude proteins, whole amino acid increased 453.47%, 10.39%, 17.76%, respectively [303].

### 5.6. Interactions Between Rapeseed and Soil Microorganisms

It is accepted that the yield and the value of *B. nepus* crop are conditioned by the rate of growth, the length of the growing period and interactions with soil microorganisms [304,305].

The effect of multifaceted, desirable or undesirable impact on microbial composition in soil is systematically increased with enlarged area of rapeseed cultivation. Several compounds identified as rapeseed components as well as their degradation products are highly biologically active and affect soil microbial community composition and soil structure [306,307]. The potential of this bio-protective agents as antifungal, antibacterial, bioherbicidal, insecticidal and nematocidal was also thoroughly documented for other members of the Brassicaceae family. Their ability to suppress soilborne disease pathogens is evaluated advantageous and surpassing the harm to beneficial biota [308,309]. The phytochemicals are released in root exudates and through decomposition of residues [310]. The biotoxic effect in soil is caused by composting seeds, meal, vegetative tissue, residual material remaining after harvesting but it is the strongest when winter canola cover crop is used as a green manure [311]. The allelopathic potential of rapeseed is so strong that it is often compared to the effects caused by chemical soil fumigation using methylbromide [312,313,314]. The main advantage of plant-derived phytochemicals are relatively fast decay leading to non-harm residual products and strongly diversified interactions with soil microbiota. As the most active group of natural pest-deterring compounds efficiently protecting Brassica species are evaluated glucosinolates. Isothiocyanates participating as active component of this plant phytochemicals are strongly sorbed by the organic matter in soil, react with nucleophilic groups present in soil, and are prone to volatilization losses, microbial degradation and mineralization minimizing the risks of persistence in the environment or leaching [308,315].

The concentrations of isothiocyanates in the soil following inclusion of Brassica residues is relatively low, due to the slow release of biologically active breakdown products what suggest that the limiting factor in this transformation is not the glucosinolate content of the plant, but rather the rate of the hydrolysis. It has to be noted that several soil microorganisms produce and excrete enzymes resulting the myrosinase activity in soil and soil extracts causing glucosinolate hydrolysis even without previous growth of Brassicaceae species [314,316]. The rate of degradation depends on soil properties. The half-life degradation of glucosinolates in a clayey soil proceeded in 3.5–6.8 h, and was much faster than in a sandy soil (half-life, 9.2–15.5 h). In the subsoil (under 25 cm soil depth), degradation was substantially slower or nonproceeded at all [317,318].

The ability to suppress soilborne disease pathogens is extremely beneficial, although the suppressing effects on mycorrhizae, rhizobia and free-living nitrogen-fixing bacteria must not to be neglected as it could cause significant harm to the beneficial biota. Moreover, fungi and arbuscular mycorrhizae fungi were found more sensitive towards Brassica then bacteria [311,316]. In fungus, isothiocyanates inhibit the oxygen uptake, the electron transport and disrupts oxidative phosphorylation in mitochondria. In bacteria, isothiocyanates inactivate various intracellular enzymes by breakdown of disulfide bridges and impede ATP synthesis in cells. In insects, isothiocyanates inactivate the thiol group of enzymes, alkylate the nucleophilic groups and increase ATP consumption by accelerating respiration, while at the same time blocking ATP production. In plants, isothiocyanates inhibit seed germination by interfering with protein synthesis and formation of phosphorylated sugars and other plant enzyme activity [309]. Applications of 10 µg of isothiocyanates to 1 g of soil reduce nitrification capacity of soil microbes by 35–65%, while addition of 0.5 µg of 2-propenyl isothiocyanate per 1 g of dry soil was sufficient to completely inhibit nitrification [319].

The microbial activity was measured by colorimetric determination of soil β-glucosidase and dehydrogenase activity [299]. Comprehensive and quantitative measurement of bacterial and fungal existence in soil can be evaluated by phospholipid analysis. This analytical method is based on the fact that bacterial and fungal cell walls are composed of phospholipids that after cell death are rapidly degrading and therefore represent a reliable measure for living microorganisms [320,321]. Another method of soil microbiome analysis is based on DNA sequencing techniques leading to their metagenomic profiling of soil ecosystem [322].

The influence on soil biological properties and crop productivity is, however, complex and crop specific. Overwintering Brassica crops are capable of capturing excess nitrate remaining after harvest, preventing nitrogen loss from leaching [310,311,323,324,325].

On the other hand, there are reports of the phytotoxic effects of the presence of Brassica residues in soil to the seedlings, causing decreased coleoptile and radicle elongation were found extracts of canola leaves [326]. The precaution is particularly important because there are well documented observations of reducing or delaying seed germination and suppressing individual plant growth by water-soluble allelochemicals leached from the mustard residues [327,328,329,330].

A biological fertilizer consisting of Bacillus megaterium A6 cultured on oilseed rape meal improved oilseed rape seed yield relative to the nontreated control in greenhouse experiments using natural soil. The fertilizer improved plant phosphorus nutrition, resulting in significantly greater available phosphorus in natural soil and in significantly greater plant phosphorus content relative to the nontreated control [331].

## 6. Conclusions

Rapeseed meal is a protein-rich byproduct with high potential as a component of animal feed, owing to its favorable amino acid profile and abundance. However, its broader use remains limited by the presence of antinutritional factors, including glucosinolates, phytic acid, synapine, erucic acid, and tannins. Although modern breeding strategies and processing technologies have reduced some of these constraints, challenges related to nutritional bioavailability, safety, and sensory quality remain. Microbial valorization offers a promising and sustainable solution to these challenges. Microorganisms and their enzymes—particularly those produced by GRAS (Generally Recognized as Safe) strains—can degrade antinutritional components, enhance protein digestibility, and convert waste into valuable bioactive compounds or feed supplements. Numerous studies have demonstrated the feasibility of such approaches, including improvements in animal health and reduced environmental impact. Despite these promising results, further research is required to address key gaps. These include strain optimization for specific transformation targets, upscaling of fermentation technologies, economic analysis, regulatory evaluation, and the stability of microbial products during feed processing and storage. Moreover, synergistic strategies involving microbial consortia or enzyme cocktails merit deeper exploration. In conclusion, microbial valorization of rapeseed oil residues represents a forward-looking strategy aligned with circular economy principles. With continued interdisciplinary research and technological innovation, this approach holds great promise for transforming agri-food waste into high-value, sustainable resources.

## Data Availability

This review article does not contain any primary data as it synthesizes and analyzes existing literature and research findings.

## Abbreviations

The following abbreviations are used in this manuscript:

EUBIA	European Biomass Industry Association
FAOSTAT	Food and Agriculture Organization Statistics
UPLC	Ultra-performance liquid chromatography
NRRL	Northern Regional Research Laboratory
